# A Proteomic Analysis of the Body Wall, Digestive Tract, and Reproductive Tract of *Brugia malayi*

**DOI:** 10.1371/journal.pntd.0004054

**Published:** 2015-09-14

**Authors:** C. Paul Morris, Sasisekhar Bennuru, Laura E. Kropp, Jesse A. Zweben, Zhaojing Meng, Rebekah T. Taylor, King Chan, Timothy D. Veenstra, Thomas B. Nutman, Edward Mitre

**Affiliations:** 1 Department of Microbiology, F. Edward Hébert School of Medicine, Uniformed Services University of the Health Sciences, Bethesda, Maryland, United States of America; 2 National Institute of Allergy and Infectious Disease, National Institutes of Health, Bethesda, Maryland, United States of America; 3 Protein Characterization Laboratory Cancer Research Technology Program, Leidos Biomedical Research inc., Frederick National Laboratory, Frederick, Maryland, United States of America; 4 Department of Biology, Frostburg State University, Frostburg, Maryland, United States of America; Washington University School of Medicine, UNITED STATES

## Abstract

Filarial worms are parasitic nematodes that cause devastating diseases such as lymphatic filariasis (LF) and onchocerciasis. Filariae are nematodes with complex anatomy including fully developed digestive tracts and reproductive organs. To better understand the basic biology of filarial parasites and to provide insights into drug targets and vaccine design, we conducted a proteomic analysis of different anatomic fractions of *Brugia malayi*, a causative agent of LF. Approximately 500 adult female *B*. *malayi* worms were dissected, and three anatomical fractions (body wall, digestive tract, and reproductive tract) were obtained. Proteins from each anatomical fraction were extracted, desalted, trypsinized, and analyzed by microcapillary reverse-phase liquid chromatography-tandem-mass spectrometry. In total, we identified 4,785 *B*. *malayi* proteins. While 1,894 were identified in all three anatomic fractions, 396 were positively identified only within the digestive tract, 114 only within the body wall, and 1,011 only within the reproductive tract. Gene set enrichment analysis revealed a bias for transporters to be present within the digestive tract, suggesting that the intestine of adult filariae is functional and important for nutrient uptake or waste removal. As expected, the body wall exhibited increased frequencies of cytoskeletal proteins, and the reproductive tract had increased frequencies of proteins involved in nuclear regulation and transcription. In assessing for possible vaccine candidates, we focused on proteins sequestered within the digestive tract, as these could possibly represent “hidden antigens” with low risk of prior allergic sensitization. We identified 106 proteins that are enriched in the digestive tract and are predicted to localize to the surface of cells in the the digestive tract. It is possible that some of these proteins are on the luminal surface and may be accessible by antibodies ingested by the worm. A subset of 27 of these proteins appear especially promising vaccine candidates as they contain significant non-cytoplasmic domains, only 1–2 transmembrane domains, and a high degree of homology to *W*. *bancrofti* and/or *O*. *volvulus*.

## Author Summary

Filarial worms are parasitic worms that can live for years within humans and cause diseases such as elephantiasis and river blindness. In this study, we identified the proteins that exist within the worm's digestive tract, reproductive tract, and body wall. In addition to increasing our understanding of the basic biology of these parasites, this information is valuable for predicting which proteins may be candidates for vaccine development and rational drug design. Specifically, by analyzing which intestinal proteins are likely expressed on the surface of cells contained within the parasite's digestive tract and have little similarity to human proteins, we identified 27 possible vaccine candidates that warrant further study.

## Introduction

*Wuchereria bancrofti* and *Brugia malayi* are filarial parasites that are the major causative agents of lymphatic filariasis (LF). Currently, it is estimated that over 129 million people are infected with either of these organisms and over one billion live in at-risk areas. Since 2000, there has been an ongoing effort through the Global Program to Eliminate Lymphatic Filariasis to eradicate these infections. While this program is having a substantive impact on the prevalence of infection, its efficacy is limited by the need to repeatedly treat entire endemic populations for 6–10 years [1, 2]. The advent of new tools, such as vaccines or more effective anthelmintics, would be of great benefit toward these eradication efforts.

The design of new tools against filariae requires a strong understanding of the parasite's biology. Recent work in genomics and proteomics has started to overcome our knowledge gaps [3–5]. The genomes of *Brugia malayi* and *Loa loa* have been published [3, 6], and the genomes of *W*. *bancrofti* and *O*. *volvulus* have also been completed (http://www.wormbase.org/tools/genome/gbrowse/o_volvulus_PRJEB513/][http://nematode.net/NN3_frontpage.cgi?navbar_selection=speciestable&subnav_selection=Wuchereria_bancrofti). Studies to identify the proteins present in life cycle stages and excretory/secretory (ES) products of *B*. *malayi* have been carried out, and key proteins in the reproductive processes have been identified [7–10]. To date, though, no inclusive study has been done on the anatomic localization of proteins in filarial worms.

**Fig 1:**
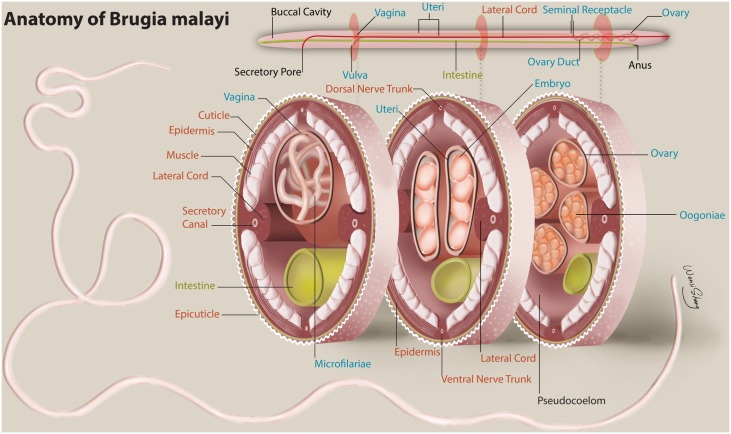
Anatomy of adult female *B*. malayi. Tissues and structures dissected for proteomic analysis include body wall (red labels), reproductive tract [blue labels], and digestive tract (green labels). Illustrated by Wensi Sheng.

Filariae are parasitic nematodes that fall within the *Metazoa* kingdom. Their anatomy is complex and includes body wall structures (cuticle, epidermis, musculature and lateral cords) as well as fully formed reproductive and digestive tracts (Figs 1 and S1). Knowledge of anatomic location of proteins within these parasites may provide information about likely physiologic function and insights regarding potential rational approaches for drug and vaccine design. Thus, in this study we performed a proteomic analysis of the digestive tract, body wall, and reproductive tract of the human filarial parasite *B*. *malayi*. With respect to vaccine design, our group was particularly interested in identifying the proteome of the filarial digestive tract as work with a number of helminths has shown potential for intestinal antigens as vaccines [11–20].

## Methods

### Dissections

**Fig 2:**
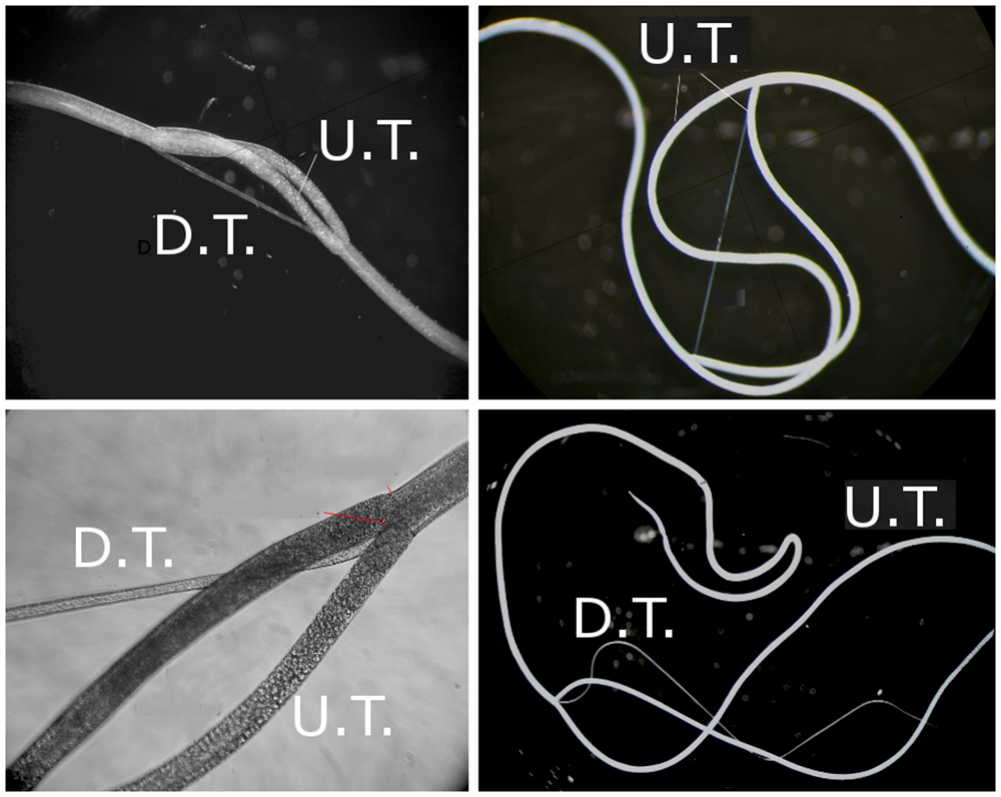
Dissection process of adult female *B*. *malayi*. Top left and bottom left show break in the body wall and extrusion of digestive and reproductive tracts. Top right and bottom right: Body wall is in process of being slid away from digestive and reproductive tracts. Magnification: top left: 40x, bottom left: 100x, top right: 30x, bottom right: 20x.

Adult *B*. *malayi* worms that were producing microfilariae were received in multiple shipments from TRS Labs (Athens, GA) and frozen at -80°C until processing. For separation of anatomic structures, worms were thawed at room temperature and then dissected using a stereomicroscope and fine tipped forceps. One set of forceps was used to grip and steady the center of the parasite after thawing and placement into a petri dish filled with phosphate buffered saline (PBS). Another set of forceps was used to grasp and gently twist the parasite close to the first set of forceps, resulting in a tear of the body wall. The cephalic tip of the body wall was then grasped and gently peeled away from the rest of the organs. The caudal portion of the body wall was then peeled away from the digestive and reproductive tracts (Fig 2). Reproductive organs were identified by their posterior junction and then separated from the digestive tract. Each anatomic fraction (digestive tract, reproductive tract, and body wall) was placed in a microcentrifuge tube filled with PBS. These were stored at -20°C until protein extraction.

### Protein extraction

The samples were thawed and then centrifuged in 1.5 ml eppendorf tubes. The pelleted tissues were frozen and thawed 4 times by cycling through placement on dry ice for 10 min. followed by placement in a 37°C water bath. Using a mini disposable micropestle, the samples were homogenized with 50 μl of UPX extraction buffer (Expedeon). The micropestle was washed with 50 μl of UPX extraction buffer and processed as per the manufacturer’s instructions. In brief, samples were placed in a 100°C water bath for 5 minutes, removed and cooled at 4°C for one hour. Samples were then centrifuged at 15,000 x g for 10 minutes, and supernatant was collected.

Protein concentrations were measured by BCA assay. 400 μg proteins of digestive tract, body wall and reproductive tract each were reduced, alkylated and trypsin digested overnight following filter-aided digestion using a FASP digestion kit (Protein Discovery, San Diego, CA) according to vendor protocol. Tryptic peptides were further desalted, lyophilized and reconstituted in 25% acetonitrile with 0.1% formic acid and further fractionated using strong cation exchange (SCX) chromatography. The SCX fractions of the three samples were collected into 16 to 18 fractions each, lyophilized and reconstituted in 0.1% trifluoroacetic acid to be analyzed by liquid chromatography-mass spectrometry (LC-MS).

### Nanobore reversed-phase liquid chromatography tandem MS (nanoRPLC-MSMS)

Nanobore RPLC-MSMS was performed using an Agilent 1200 nanoflow LC system coupled online with a LTQ Orbitrap XL mass spectrometer. The RPLC column (75 μm i.d. x 10 cm) were slurry-packed in-house with 5 μm, 300Å pore size C-18 stationary phase into fused silica capillaries with a flame pulled tip. After sample injection, the column was washed for 20 min with 98% mobile phase A (0.1% formic acid in water) at 0.5 μl/min. Peptides were eluted using a linear gradient of 2% mobile phase B (0.1% formic acid in acetonitrile) to 35% B in 100 minutes, then to 80% B over an additional 40 minutes. The column flow-rate was maintained at 0.25 μl/min throughout the separation gradient. The mass spectrometer was operated in a data-dependent mode in which each full MS scan was followed by seven MS/MS scans wherein the seven most abundant molecular ions were dynamically selected for collision-induced dissociation (CID) using a normalized collision energy of 35%.

### Protein identification

The LC-MS/MS data were searched using SEQUEST through Bioworks interface against a combined database of *Brugia malayi* database downloaded from The Institute for Genomic Research (TIGR) (updated 12/21/2006), and the *Wolbachia* database from New England Biolabs (Beverly, MA). Carbamidomethyl of cysteine was specified in Sequest as a fixed modification. Oxidation of methionine was specified in Sequest as a variable modification. Scaffold (version Scaffold_3.5.2, Proteome Software Inc., Portland, OR) was used to validate MS/MS based peptide and protein identifications. Peptide identifications were accepted if they could be established at greater than 95.0% probability by the Peptide Prophet algorithm [21]. Protein identifications were accepted if they could be established at greater than 99.0% probability and contained at least 2 identified peptides. Protein probabilities were assigned by the Protein Prophet algorithm [22]. Proteins that contained similar peptides and could not be differentiated based on MS/MS analysis alone were grouped to satisfy the principles of parsimony. TIGR accession numbers were matched to PUB_loci from the proteome published by Bennuru et. al. [4]. Proteins were also matched by amino acid sequence to the wormbase WS230 version (2012) of the *Brugia malayi* proteome.

### Quantitative analysis

Protein quantitation was determined by normalized spectral abundance. This approach provides a theoretical quantitative value useful for determining relative abundance of a single protein among samples [23, 24] and an estimation of relative abundance among different proteins in one sample [25]. Exclusive spectral counts, spectra that match to only 1 protein, were first divided by the length of the protein to account for the differences in numbers of possible spectra. This calculation provides the spectral abundance factor. This was then normalized to obtain the normalized spectral abundance factor (NSAF) by dividing by the sum of the total spectral abundance factors found within that anatomic fraction.$\text{NSAF}=\frac{{\left(\frac{\text{Spectra}}{\text{Length}}\right)}_{\text{p}}}{\sum_{\text{p}=1}^{\text{n}}{\left(\frac{\text{Spectra}}{\text{Length}}\right)}_{\text{p}}}$. NSAF enrichment was then calculated by dividing the NSAF of a given protein in the target fraction divided by the sum of the NSAF of the other two fractions to determine whether a protein was more abundant or "enriched" in one fraction compared to the others.$\text{NSAF enrichment}=\frac{\text{NSAF}(\text{target fraction})}{\text{NSAF of other two fractions }(\text{added})}$. Proteins with NSAF enrichment values of 2 or greater were were considered enriched within that fraction.

### Functional categories for gene set enrichment analysis (GSEA)

The proteome of *B*. *malayi* had previously been functionally characterized by Bennuru and colleagues [4]. For proteins previously annotated for function, no further analysis of function was carried out. The 665 newly identified proteins were annotated based loosely on the eukaryotic orthologous groups (KOG) and protein family (PFAM) functions. Categories of function were used as previously described [4], including cytoskeletal, extracellular matrix, immunological, metabolism, nuclear regulation, protein export, protein modification, protein synthesis, signal transduction, transcription, transporters, and uncharacterized. Functions of anatomic fractions were analyzed based on GSEA, which analyzes the data for bias in a condition (or anatomic fraction) [26]. Proteins were ranked according to abundance using spectral counts. A priori defined sets of proteins, based on functional annotation, were then analyzed using GSEA for bias within each anatomic fraction. All categories of proteins were analyzed for bias within each anatomic fraction. Only those categories which showed significant bias for a fraction are discussed.

### Histology

Live *B*. *malayi* worms were quick-frozen in cold isopentane and OCT compound. Cryosections of 6 microns thickness were cut with a cryostat and fixed in cold 70% EtOH (for H&E staining) or cold acetone (for actin/DAPI staining) for 10 minutes. Acetone-fixed sections were washed in PBS, blocked with 1% BSA for 30 minutes, and stained with a mixture of DAPI (Sigma) and ActinGreen 488 ReadyProbes Reagent (Molecular Probes). DAPI was used at 300nM and the ActinGreen Reagent was used as directed. Sections were washed in PBS, mounted with Fluoromount-G (eBioscience) and imaged on a Nikon E600 microscope with Nikon Elements software.

### BLASTp

BLASTp was performed on proteins of interest from *B*. *malayi* to identify similarity among *W*. *bancrofti*, *O*. *volvulus*, *D*. *immitis*, *L*. *loa*, and *H*. *sapiens*. BLAST query was conducted with blast+ 2.2.29 downloaded from NCBI at ftp://ftp.ncbi.nlm.nih.gov/blast/executables/blast+/LATEST/. Protein databases for *W*. *bancrofti*, *O*. *volvulus*, *H*. *sapiens*, *L*. *loa* were downloaded from uniprotKB. Protein database for *D*. *immitis* was downloaded from www.nematodes.org. A FASTA file containing the *B*. *malayi* proteins of interest were blasted against each of the other genomes individually. Percent identity and query coverage were recorded for the top scoring sequence for each protein. Score is determined by an algorithm that takes into account similarity of AA sequence, gaps in homologous regions, and length of homology. Percent identity is defined as the percentage of amino acids that match perfectly over the sequence region with greatest homology.

## Results

### Distinct anatomic fractions exhibit markedly different expression of proteins

**Fig 3:**
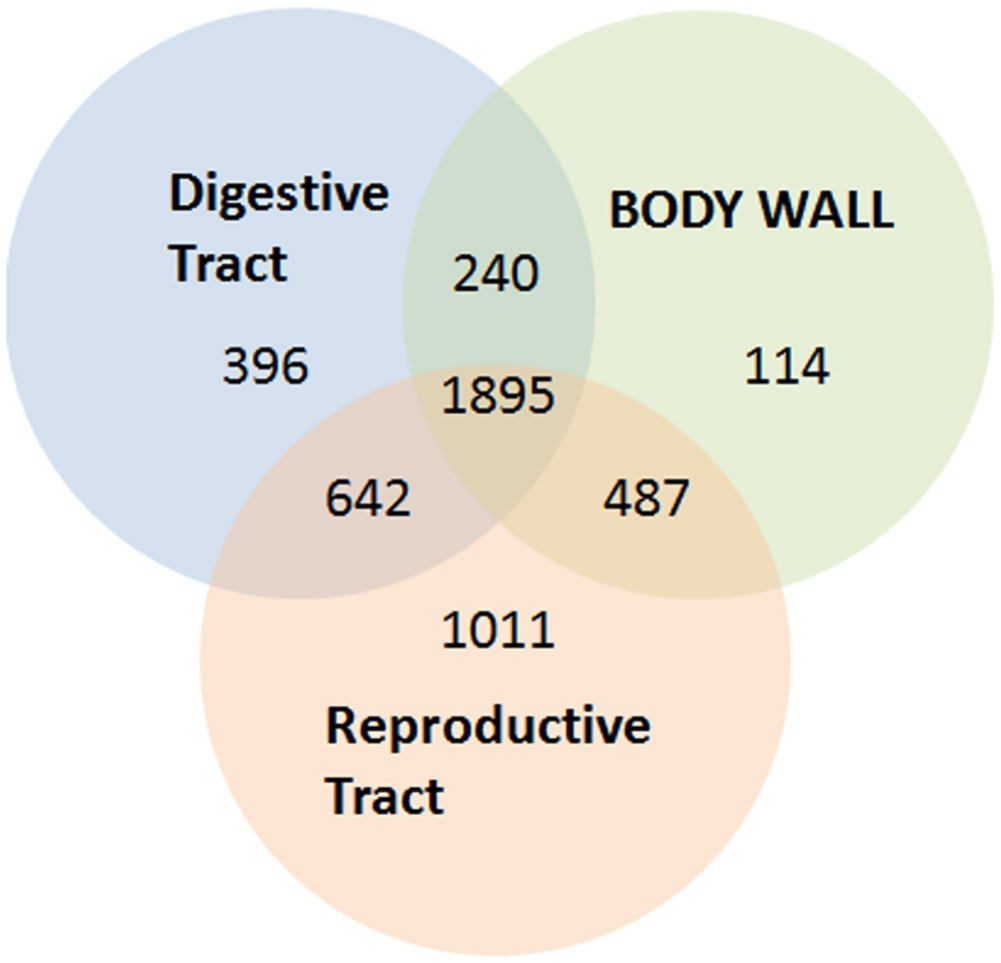
Venn diagram of proteins identified within each anatomic fraction of adult female *Brugia malayi* based on 2 peptide minimum for identification.

Based on a minimum match of 2 unique peptides to a protein, we identified a total of 4,989 proteins. Of these, 4,785 were identified as *B*. *malayi* proteins (S1 Dataset), with the remaining 204 being *Wolbachia* proteins (S2 Dataset). Of the *B*. *malayi* proteins, 1,895 were identified by at least two peptides in all three anatomical fractions of the parasite, 396 proteins were identified solely within the digestive tract, 114 solely within the body wall, and 1011 solely within the reproductive tract (Fig 3).

### Proteomic profiling of the *B*. *malayi* intestine is consistent with functional absorption and digestion

**Fig 4:**
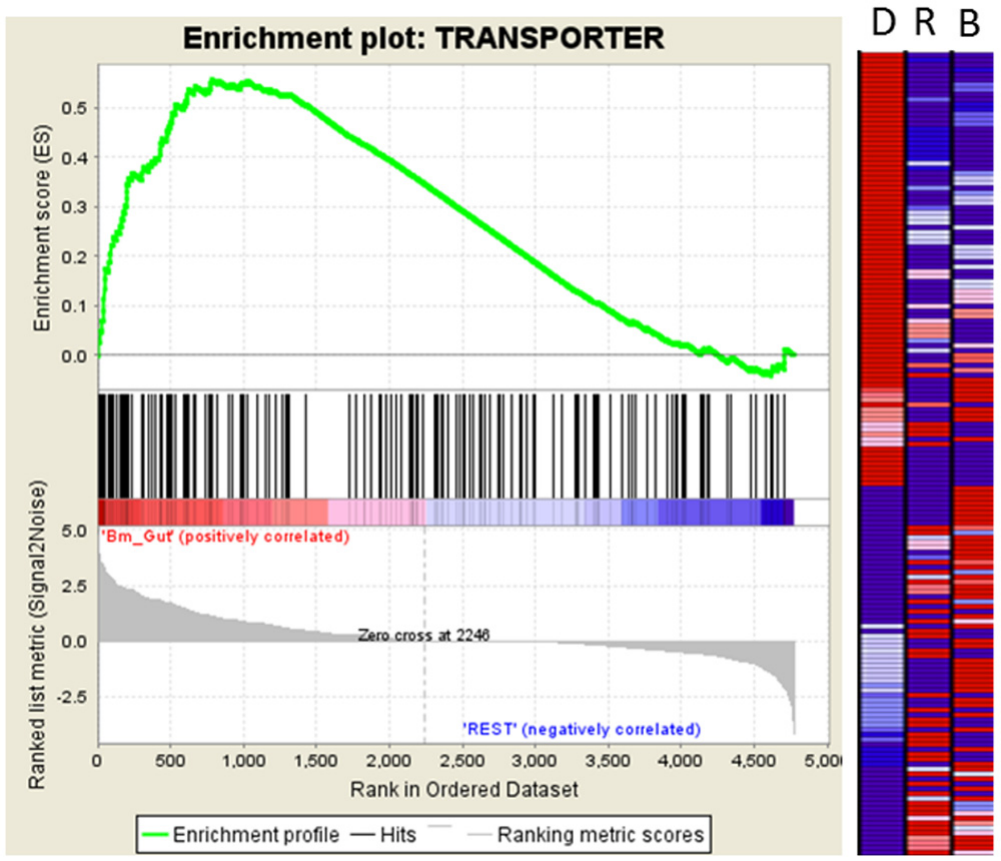
Association of transporter proteins with the digestive tract as measured by gene set enrichment analysis (GSEA). P-value = 0.005. The enrichment score is represented by the green line. Proteins were rank ordered according to their number of spectral counts within the digestive tract and are depicted in the heat map (red = more abundant, blue = less abundant). Black vertical lines represent each of the proteins associated with transporter function. D = Digestive tract, R = Reproductive tract, and B = Body wall.

Like most nematodes, filarial parasites have a fully formed digestive tract. However, the functionality of this tract is not completely clear [27]. We performed several analyses to further elucidate thefunction of the digestive tract in *B*. *malayi*. First, gene set enrichment analysis (GSEA) was performed which showed a bias for proteins with transporter function to be present within the digestive tract (Fig 4). Next, we rank ordered the proteins that were enriched within the digestive tract based on their NSAF value, a measure which takes into account the number of spectra uniquely matching to a protein and the length of the protein in amino acids. Spectral counting has previously been shown to be useful to determine relative abundance of a single protein in different samples [23, 24] and provide a reasonable approximation of protein abundance within a sample compared to other proteins in the same sample [25]. Of the 20 most abundant, enriched, and named digestive tract proteins, 3 are proteolytic enzymes (Bm1_00205, Bm1_18805, Bm1_34740), 2 are transporters (Bm1_42930, and Bm1_24840), and 1 is associated with phagocytosis (Bm1_02265). The abundance of such proteins suggests the digestive tract may be involved in both digestion and active absorption of nutrients. Of the remaining 20 most abundant named proteins in the digestive tract, 3 are muscle associated proteins (Bm1_28910, Bm1_45035, Bm1_00655) and the rest are involved in various functions including translation, cell trafficking, RNA binding, cell adhesion, hydrolysis, lipid metabolism, catabolism, and cellular structure (Table 1).

**Table 1:** 20 most abundant proteins, with proper names, enriched in the digestive tract of adult female *B*. *malayi* based on normalized spectral abundance factor (NSAF).

			Abundance NSAF	NSAF enrichment
Protein Type	Accession	Name	Digestive tract	Digestive tract
Translational	Bm1_41515	40S ribosomal protein S21, putative	5.4E-03	2.17
Muscle Associated	Bm1_28910	Calsequestrin, skeletal muscle isoform precursor, putative	2.5E-03	2.90
Cell trafficking	Bm1_14235	SNARE domain containing protein	2.4E-03	2.0
Muscle Associated	Bm1_45035	Probable myosin regulatory light chain, putative	2.2E-03	2.5
Protease	Bm1_34740	aspartic protease BmAsp-1, identical	1.1E-03	16.0
carrier protein	Bm1_21135	Acyl CoA binding protein	9.0E-04	7.68
Muscle Associated	Bm1_00655	myosin heavy chain, nonmuscle type 1, putative	7.8E-04	2.0
Phagocytosis associated	Bm1_02265	MGC69076 protein-related	7.3E-04	3.77
Xenobiotic metabolism	Bm1_13480	UDP-glucoronosyl and UDP-glucosyl transferase family protein	7.0E-04	28.16
RNA binding	Bm1_20295	Glycine-rich RNA-binding protein.-related	6.9E-04	8.96
Miscellaneous	Bm1_25280	Prion-like—related	6.4E-04	2.37
Cell Adhesion	Bm1_10500	AMOP domain containing protein	6.1E-04	5.99
Hydrolase	Bm1_24820	Histidine acid phosphatase family protein	6.1E-04	6.32
Cytoskeleton	Bm1_30265	Tubulin alpha chain, putative	5.9E-04	2.95
Transporter	Bm1_42930	Excitatory amino acid transporter, putative	5.7E-04	2.75
Lipid Metabolism	Bm1_08150	NAD-dependent malic enzyme, mitochondrial precursor, putative	5.5E-04	7.10
Catabolism	Bm1_48185	putative amidase	5.1E-04	3.74
Transporter	Bm1_24840	Major Facilitator Superfamily protein	4.7E-04	19.88
Protease	Bm1_18805	Papain family cysteine protease containing protein	4.4E-04	*
Protease	Bm1_00205	ShTK domain containing protein	4.2E-04	3.42

* Protein was only found within the digestive tract.

### Many predominant body wall enriched proteins provide muscular structure or are involved in muscular contraction

The body wall of *B*. *malayi* includes, from superficial to deep, the epicuticle, cuticle, epidermis, musculature (divided into a superficial fibrous portion and a deeper metabolically active portion) and the lateral cords (Figs 1 and S4). The musculature is separated into quadrants by the lateral, ventral and dorsal cords with up to 9 myocytes per quadrant [28]. The lateral cords contain the cell bodies of the epidermis, which produces and maintains the cuticle. Also associated with the lateral cords is a secretory gland, which is connected to the secretory pore by the secretory canal [29]. The ventral and dorsal cords are associated with nerves that innervate the musculature.

GSEA of the body wall showed a bias for cytoskeletal proteins and proteins of immunological interest to be present within the body wall (S2 Fig). Further, analysis of the 20 most abundant named proteins that were enriched within the body wall by NSAF yielded 12 proteins associated with muscle structure or regulation of muscular contraction (Table 2). These included an actin (Bm1_21705), 4 myosins (Bm1_40715, Bm1_50805, Bm1_00935, Bm1_14060), 2 paramyosins (Bm1_04450, Bm1 02615), 1 tropomyosin (Bm1_02060), and a disorganized muscle protein (Bm1_40320). In addition to the muscular proteins, there were 3 cuticular proteins, a glutathione peroxidase, which likely provides protection from oxidative damage, a cytoskeletal protein, a heat shock protein, and a glutamine synthetase (Table 2).

**Table 2:** 20 Most abundant proteins, with proper names, enriched in the body wall of adult female *Brugia malayi*.

			Abundance (NSAF)	NSAF enrichment
Protein type	Accession	Name	Body Wall	Body Wall
Muscle associated	Bm1_21705	actin 1, putative	6.2E-02	3.86
Cytoskeletal	Bm1_45215	intermediate filament protein, putative	2.0E-02	2.94
Muscle associated	Bm1_40320	Disorganized muscle protein 1, putative	1.6E-02	5.15
HSP	Bm1_19805	small heat shock protein, putative	1.4E-02	7.97
Muscle associated	Bm1_04450	Paramyosin, putative	1.1E-02	5.87
Muscle associated	Bm1_02615	Paramyosin, identical	1.0E-02	6.33
Calcium Binding	Bm1_48810	EF hand family protein	6.5E-03	9.81
Cuticle	Bm1_13015	Nematode cuticle collagen N-terminal domain containing protein	6.1E-03	3.05
Muscle associated	Bm1_01235	Tropomyosin-related	6.0E-03	5.24
Muscle Associated	Bm1_49075	Calponin homolog OV9M, putative	5.9E-03	3.31
Muscle associated	Bm1_40715	myosin heavy chain, putative	5.8E-03	3.11
Cuticle	Bm1_54705	Nematode cuticle collagen N-terminal domain containing protein	5.5E-03	6.57
Muscle associated	Bm1_50805	Myosin tail family protein	4.7E-03	4.00
Antioxidant	Bm1_40465	Cuticular glutathione peroxidase precursor, putative	4.5E-03	2.59
Muscle associated	Bm1_00935	myosin heavy chain B (MHC B), putative	4.4E-03	3.36
Carbohydrate metabolism	Bm1_16060	carbohydrate phosphorylase, putative	4.2E-03	2.67
Muscle associated	Bm1_14060	myosin heavy chain B (MHC B), putative	4.1E-03	2.23
Cuticle	Bm1_17485	Nematode cuticle collagen N-terminal domain containing protein	3.2E-03	2.40
Muscle associated	Bm1_02060	Tropomyosin family protein	3.2E-03	3.19
Amino Acid Synthesis	Bm1_53470	glutamine synthetase, putative	3.0E-03	3.16

### Nuclear regulatory proteins, including those involved in chromatin organization are enriched and highly abundant in the reproductive tract

The nematode female reproductive tract consists of two ovaries where gamete production takes place, two seminal receptacles (aka spermatheca), which store sperm obtained from males, and 2 uterine tubes that allow for embryo and subsequent *in utero* microfilaria development (Fig 1). The two uterine tubes merge into the vulva, which is on the ventral surface of the worm in the cephalic region [7, 9, 29, 30]. GSEA showed a bias for transcription and nuclear regulation proteins to be present within the female reproductive tract (S3 Fig). Similarly the 20 most abundant named proteins that were enriched in the reproductive tract as assessed by NSAF contained many proteins involved in nuclear regulation. 12 proteins contained domains associated with nucleotide binding or splicing, with 8 of these 12 being histones or histone linkers (Bm1_02505, Bm1_02515, Bm1_20280, Bm1_02495, Bm1_, 20285, Bm1_, 38685, Bm1_02800, Bm1_04110) (Table 3). 3 microfilarial sheath proteins were also abundant and enriched within the reproductive tract, which is consistent with presence of developing microfilariae within the uterine tubes. The remaining 7 proteins are involved in trafficking, protection from oxidation, xenobiotic metabolism, proteolysis and cell adhesion.

**Table 3:** 20 Most abundant proteins, with proper names, enriched in the reproductive tract of adult female *Brugia malayi*.

			Abundance NSAF	NSAF Enrichment
Protein Type			Reproductive tract	Reproductive tract
Chromatin organization	Bm1_02505	histone H2A, putative	3.4E-02	8.1
Chromatin organization	Bm1_02515	histone H4, putative	3.1E-02	2.7
Chromatin organization	Bm1_20280	Probable histone H2B 3, putative	1.1E-02	4.3
Chromatin organization	Bm1_02495	histone H3, putative	7.8E-03	25.9
Sheath	Bm1_19100	Major microfilarial sheath protein precursor.-related	6.2E-03	2.6
Chromatin organization	Bm1_20285	histone H2A, putative	5.9E-03	17.2
Chromatin organization	Bm1_38685	Histone H2A variant, putative	3.4E-03	2.7
Sheath	Bm1_05185	sheath protein 5, identical	2.8E-03	2.9
Trafficking	Bm1_07925	peroxisomal membrane anchor protein, putative	2.2E-03	2.6
Antioxidant	Bm1_44840	Glutathione S-transferase, N-terminal domain containing protein	2.0E-03	2.3
DNA binding	Bm1_25620	high mobility group protein, putative	1.7E-03	10.2
Sheath	Bm1_00650	microfilarial sheath protein, identical	1.2E-03	2.4
RNA splicing	Bm1_49560	NOP5/NOP58, putative	1.2E-03	2.2
RNA modulation	Bm1_49460	small nuclear ribonucleoprotein-associated protein homolog F9F13.90—Arabidopsis thaliana, putative	1.1E-03	2.3
Chromatin organization	Bm1_57630	retinoblastoma-binding protein., putative	1.1E-03	2.6
Chromatin organization	Bm1_04110	linker histone H1 and H5 family protein	9.6E-04	4.7
Xenobiotic metabolism	Bm1_32235	Flavin-binding monooxygenase-like family protein	9.4E-04	2.6
Chromatin organization	Bm1_02800	Histone H2B 2, putative	9.0E-04	*
Protease	Bm1_45620	Trypsin family protein	8.9E-04	36.5
Cell Adhesion	Bm1_17270	Fasciclin domain containing protein	8.1E-04	2.8

*Protein was only found within the reproductive tract.

### Identification of potential digestive tract vaccine candidates

In order to identify intestinal proteins that could potentially be used as vaccine candidates, we analyzed our protein set for cell surface digestive tract proteins, which may be accessible to host antibodies after vaccination. We identified 106 proteins likely to be present on the cell membrane, based on the criteria of being enriched in the digestive tract, having at least one transmembrane domain [4] and not being predicted to be in the mitochondria based on targetP prediction. Filtering the data for similarity with other filariae (*W*. *bancrofti*, *O*. *volvulus*, *L*. *loa*, and the dog heartworm *D*. *immitis)* and human host resulted in 72 potential vaccine candidates that exhibited ≥75% identity with *W*. *bancrofti* and *O*. *volvulus* and <40% homology to humans.

We then selected those proteins that had 1–2 transmembrane domains for ease of recombinant protein production. These were evaluated with Interpro software for the presence of non-cytoplasmic domains that could be bound by host antibodies. 27 proteins matched all of these criteria (Table 4), with 12 displaying high homology among all of the filarial species (marked with * on Table 4). Of these 27 proteins, 10 are hypothetical proteins, 3–4 are proteases, 2 are involved in xenobiotic metabolism using glucuronidation, 2 participate in cell adhesion, 2 function in cell signaling, and 2 are chaperones.

**Table 4:** Selected proteins from S1 Table that contain 1–2 transmembrane domains, a significant non-cytoplasmic portion, >75% homology to either *W*. *bancrofti*, or *O*. *volvulus*, and <40% homology to humans.

		*H*. *sapiens*		*W*. *bancrofti*		*O*. *volvulus*		*L*. *loa*		*D*. *immitis*			
Pub Locus		%Ident	Query cov.	%Ident	Query Cov	%Ident	Query Cov	%Ident	Query Cov	%Ident	Query Cov	Non-cytoplasmic domain	Transmembrane domain
**Cell Adhesion**													
*Bm1_39630	Immunoglobulin I-set domain containing protein	28	45–1170	97	628–1171	87	26–1171	89	1–1171	82	1–1171	19–1120	1
*Bm1_02820	EGF-like domain containing protein	35	53–206	96	1–269	82	3–269	89	62–269	79	2–269	1–225	1
**Cell Signaling**													
*Bm1_30585	Tyrosine-protein kinase abl-1.-related	24	212–281	95	10–281	79	10–281	83	10–281	84	10–281	19–135	1
*Bm1_19395	Protein kinase domain containing protein	34	15–1280	98	1–681	92	1–1280	95	1–1280	93	1–1280	1–942	1
*Bm1_38285	Ser/Thr protein phosphatase family protein	40	22–287	95	9–293	79	2–293	88	9–293	79	1–290	41–293	1
**Chaperone/HSP**													
*Bm1_15660	DnaJ domain containing protein	27	25–835	91	1–839	77	3–839	85	11–839	80	1–839	18–220	1
*Bm1_22450	hemimethylated DNA binding domain containing protein	25	34–112	97	29–119	95	29–119	97	29–119	91	28–119	1–125	1
**Glycosylation/glucuronidation**													
#Bm1_44655	Fukutin.-related	31	138–364	96	1–362	73	1–364	85	1–364	75	1–364	28–364	1
#Bm1_13480	UDP-glucoronosyl and UDP-glucosyl transferase family protein	27	35–509	95	1–425	29	214–293	81	1–423	72	155–502	1–486	1
**Miscellaneous**													
#Bm1_49590	CG3054-PA-related	28	97–260	81	1–242	69	1–260	63	1–262	67	1–254	1–51, 101–265	2
*Bm1_10500	AMOP domain containing protein	26	652–932	98	679–1377	92	1–1513	94	1–1513	90	609–1513	23–1322	1
#Bm1_48010	EGF-like domain containing protein	36	10–395	91	19–338	66	6–560	74	6–556	64	6–546	1–430	1
**Proteases**													
#Bm1_38300	Peptidase family M1 containing protein	28	136–586	90	90–819	67	1–819	74	1–819	70	1–819	81–1061	1
*Bm1_11005	MGC84665 protein-related	38	2–98	95	1–96	80	1–98	91	1–98	90	1–40	1–16,76–169	1
*Bm1_53050	Reprolysin	32	153–753	91	96–845	77	1–839	79	1–839	77	1–843	1–607	1
**Possible Proteases**													
#Bm1_00205	ShTK domain containing protein	27	54–161	80	142–229	52	70–227	46	126–264	55	110–264	26–264	1
**Protease Inhibitors**													
#Bm1_09775	serpin, putative	29	26–388	75	84–375	52	29–390	54	1–391	52	1–391	17–391	1
**Hypothetical Proteins**													
*Bm1_52210	hypothetical protein	29	238–350	97	53–433	85	1–431	87	15–432	84	1–430	1–369	1
*Bm1_57335	Conserved hypothetical protein, putative	31	114–236	96	1–246	89	1–246	91	1–246	87	1–246	29–211	2
$ &Bm1_20460	hypothetical protein	30	103–147	24	94–147	82	1–191	86	1–191	85	1–191	143–147	2
#Bm1_45100	hypothetical protein	29	330–414	85	118–727	60	75–727	67	81–696	49	271–727	285–727	1
* &Bm1_07875	CONSERVED HYPOTHETICAL PROTEIN	31	84–225	99	142–231	85	53–231	90	42–231	90	41–231	1–56.	1
*Bm1_17550	hypothetical protein	33	63–121	87	77–129	82	82–125	77	77–129	86	82–125	1–61.	1
#Bm1_07845	hypothetical protein	28	24–108	89	1–210	64	1–210	75	1–210	72	60–210	53–210	1
^Bm1_17255	hypothetical protein	21	93–246	87	17–251	78	28–251	73	13–251	75	28–251	36–251	1
*Bm1_20325	Hypothetical protein-conserved	37	2–215	96	1–487	89	1–487	91	1–487	89	1–485	1–194	1
#Bm1_46230	hypothetical protein	29	184–256	91	24–278	62	1–278	65	18–278	31	161–281	121–289	1

Non-cytoplasmic domain refers to the span of amino acids predicted to be non-cytoplasmic.

^#^ >75% homology to *W*. *bancrofti*,

^$^ >75% homology to *O*. *volvulus*,

^^^ >75%homology to both *W*. *bancrofti* and *O*. *volvulus*,

*>75% homology to *W*. *bancrofti*, *O*. *volvulus*, *L*. *loa*, and *D*. *immitis*.

^&^Previously found in the excretory/secretory products of adult female *B*. *malayi*

### Certain excretory/secretory (ES) products are associated with specific anatomic fractions

Previously, Bennuru and colleagues identified 227 proteins excreted and/or secreted by adult female *B*. *malayi* [5]. To better define the origin of these proteins, we analyzed all adult female excreted/secreted (ES) proteins for enrichment within any of the three worm fractions from this study. 4 (1.7%) of these proteins were either enriched or specific to the digestive tract (S2 Table). The most notable of these was the papain family cysteine protease (Bm1_18805). Eight (3.5%) ES products were enriched within the body wall (S3 Table), including two proteins that protect against oxidative damage, cuticular glutathione peroxidase (Bm1_40465) and peptide methionine sulfoxide reductase (Bm1_10795) [31]. Other ES products enriched within the body wall included a cuticle collagen (Bm_13015), and muscular proteins.

There were 30 adult female ES products (13%) enriched within the female reproductive tract (S4 Table). Some of these antigens include Juv-p120 (Bm1_18010), which has been implicated as being critical for MF survival, Von willebrand factor type A domain containing protein (Bm1_27495), which likely binds to collagen, a trypsin inhibitor (Bm1_03520), and an aspartyl amino peptidase (Bm1_16690).

## Discussion

This study assessed the proteome of different anatomical areas of *Brugia malayi* to further our understanding of the biology of filarial nematodes and to potentially identify novel vaccine antigens or drug targets within the filarial digestive tract. To accomplish this, adult female *B*. *malayi* were dissected into fractions containing the digestive tract, the reproductive tract, or the body wall. These fractions went through a protein extraction process, and proteins were identified by RPLC-MS/MS. Because microdissection techniques and the use of frozen worms may have allowed some cross contamination between anatomic fractions, in the discussion we focus on the enrichment of proteins in one fraction compared to the others rather than simply evaluating the presence or absence of proteins. Enrichment analysis validated the experimental design, as the presence of many cytoskeletal proteins within the body wall and many proteins with actions on nucleic acids within the reproductive tract are highly consistent with our understanding of *B*. *malayi*.

In terms of biological insights, although it is still unclear whether the digestive tract is the primary source of nutrient uptake, this study suggests that the digestive tract of adult filarial nematodes is likely involved in at least some nutrient digestion and absorption. While such a function may seem to be an obvious one for the digestive tract of any organism, several factors in the biology of filariae have led researchers to question the role of the digestive tract in these nematodes [27]. Given that the causative agents of LF live within the lymphatics of their host and are bathed in fluid with a composition similar to plasma, there is likely little need for complex digestive processes by these parasites. It is thus unsurprising that the digestive tract of *B*. *malayi* is histologically very simple when compared to parasites such as *N*. *americanus* or *A*. *suum* that would require more substantial digestive processes based on their location within the host. Indeed, much of the digestive tract is composed of simple cuboidal epithelium, and a complete mouth to anus digestive tract is only present in the L4 and adult stages of filariae. In the microfilariae, L2, and L3 stages the digestive tract is either not present or is not fully formed [32]. These findings in combination with *Brugia's* ability to absorb nucleotides, amino acids, small peptides, sugars and vitamins directly across the cuticle have driven the skepticism of the role for a digestive tract in *Brugia malayi* [27, 32].

In contrast, GSEA analysis in this study showed a bias for transporters to be present within the digestive tract, which is consistent with the digestive tract being involved in either absorption of nutrients or removal of waste products. This is similar to what was found in a recent study analyzing differences in anatomic gene expression of the parasite of swine *A*. *suum*, which revealed elevated expression of eleven GO terms associated with transport activity within the intestine relative to other anatomic locations [33]. One major difference, however, is that their study also showed elevated expression of many types of hydrolases (including some classes of proteolytic enzymes) within the intestines of *A*. *suum*. While we did not specifically examine classes of proteolytic enzymes by GSEA for significant enrichment within any anatomic fraction., the presence of 3 proteolytic enzymes (Bm1_34740, Bm1_18805, and Bm1_00205), 2 transporters, and a protein involved in phagocytosis within the 20 most abundant named proteins of the digestive tract by NSAF analysis may suggest a role in digestion and absorption.

Interestingly, 2 of the top 20 most abundant named proteins in the digestive tract were myosin heavy chain (nonmuscle type 1, putatative, Bm1_00655) and myosin regulatory light chain (putatitve, Bm1_45035). As muscle is sparse within the nematode digestive tract and located solely within the pharynx and anus, the abundance of these proteins suggests that these proteins are not muscle derived. Instead, they are likely directly involved in the function of the filarial intestinal epithelium. In mammalian digestive tracts, proteins such as nonmuscle myosin and myosin regulatory light chain contribute to tight junction control and epithelial cell motility [34–36]. The presence of such proteins in the digestive tract of *B*. *malayi* suggests the filarial digestive tract is a dynamic organ with the capacity to perform wound repair and to calibrate barrier function. A third muscle associated protein identified as highly abundant in the *B*. *malayi* digestive tract was calsequestrin (Bm1_28910). While calsequestrin is most commonly found in the sarcoplasmic reticulum of skeletal and cardiac muscle cells, where it functions as a major calcium binding protein, calsequestrin-like proteins have also been identified in nerve cells and hepatocytes [37]. Although the role of calsequestrin in non-contractile tissues remains unknown, its abundance in *B*. *malayi* digestive tract suggests intracellular calcium storage may be important for the function of this organ in filariae.

In other nematode models, the intestine has been implicated in stress response, detoxification, reproduction, and immunity to microbes [38, 39]. Specifically, UDP transferases, major facilitator superfamily proteins, and ABC transporters have been implicated as likely having roles in xenobiotic metabolism and removal [38, 40]. Our results similarly showed high levels of proteins such as UDP-glucoronosyl and UDP-glucosyl transferase family protein (Bm_13480), the major facilitator superfamily protein (Bm1_24840), and ABC transporters within the digestive tract, which suggests that the digestive tract of *B*. *malayi* is similarly involved in detoxification and removal of xenobiotics. Given the prevalence of these detoxifying proteins, this physiologic role may be a prime target for vaccine or drug development.

Finally, our results suggest that the filarial intestine may also be involved in some yet unstudied function of worm physiology. Many of the most abundant digestive tract enriched proteins were hypothetical proteins. Indeed, the 20^th^ most abundant named protein enriched in the digestive tract was actually the 43^rd^ most abundant protein when unnamed (i.e. “hypothetical”) proteins were included in the evaluation (S5 Table). In contrast, the 20^th^ most common named protein in the body wall and reproductive tract represented the 21st and 23rd most abundant proteins in those fractions when hypothetical proteins were included (S6 and S7 Tables). Considering the high number of hypothetical proteins among the most abundant digestive tract enriched proteins, the intestinal tract may fulfill roles in parasite physiology that we do not yet understand. Based on our findings, it is likely that the intestine has a non-redundant role in the physiology of *B*. *malayi*, validating the plausibility of inducing protective immune responses by vaccination with intestinal antigens.

In contrast to the digestive tract, there has been little mystery in regards to the roles of the other two anatomic fractions, and the results of this study are consistent with our knowledge of both of these anatomic fractions. Sheath proteins and proteins that are involved in chromatin organization make up a large portion of the most abundant proteins enriched within the reproductive tract. The high level of cellular replication within the reproductive tract compared to the other anatomic fractions is clearly consistent with the abundance of histones and histone linkers that would be required for chromatin organization.

The body wall is also a well studied portion of the parasite. The deepest portion of the body wall is a layer of muscle, which contains the preponderance of *B*. *malayi* muscular tissue. As expected, the majority of the 20 most abundant named proteins that were enriched within the body wall were muscle associated proteins. The majority of these were structural proteins such as Actin (Bm1_2705), Myosin (Bm1_00935, Bm1_14060, Bm1_50805, Bm1_40715), Paramyosin (Bm1_04450, Bm1 02615) and tropomyosin (Bm1_39425). The presence of glutathione peroxidase within the body wall is notable as the cuticle of the body wall of filariae has been known to contain glutathione peroxidase, which presumably protects against leukocyte derived reactive oxygen species [41].

One of the principle obstacles in designing vaccines against helminths is that previously exposed individuals frequently have IgE to surface and secreted helminth antigens, putting them at risk for allergic reactions when re-exposed to these antigens. This was demonstrated vividly in a phase I trial evaluating Na-ASP-2, a major secreted protein of hookworm larvae, as a hookworm vaccine in humans [42]. This study had to be halted because of IgE-mediated adverse events among people who had previous exposure to hookworm.

Because of this risk of prior allergic sensitization, there is currently interest in using intestinal antigens as vaccine candidates because these antigens may not induce strong immune responses during natural infection. Prior work with a number of helminths has shown potential for intestinal antigens as vaccine candidates [11–14, 16–20]. Although our laboratory did not observe protection when vaccinating mice with a preparation of soluble *L*. *sigmodontis* intestinal antigens [43], vaccination with intestinal antigens has shown efficacy in a non-permissive Dirofilaria mouse model [44]. A major limitation of these trials was the use of crude homogenates of digestive tracts containing thousands of antigens. Vaccination with individual or small numbers of specific intestinal antigens may be more effective than vaccinating with such mixtures.

To identify potential vaccine candidates from filarial digestive tract, we screened digestive tract enriched proteins for significant extracellular domains, low homology to humans, and high homology to either *W*. *bancrofti* or *O*. *volvulus*, the major filarial pathogens of humans, and 1–2 transmembrane domains for ease of protein production. Although some of the 27 proteins that met these criteria may localize to a cell surface other than the luminal surface, it is likely that some of these proteins would be accessible to host antibodies after vaccination. Since the most promising vaccines that target the digestive tract of other helminths have been proteolytic enzymes [11–18], the proteases in this group (Bm1_38300, Bm1_11005, Bm1_53050), and the possible protease (Bm1_00205), may be prime targets for further vaccine research.

In addition to the intestinal proteases, a few other intestinal proteins may make excellent vaccine targets. Fukutin (Bm1_44655) and the UDP-glucuronosyl and UDP glucosyl transferase (Bm1_13480) have functions that could theoretically be inhibited by antibodies. UDP-glucuronosyl and UDP glucosyl transferase, for example, has a similar physiologic role to glutathione-S-transferase, an enzyme which has been shown to confer protection when used as a vaccine in animal models of filariasis [45, 46]. Both of these enzymes are involved in phase II detoxification of xenobiotics, and therefore disabling this enzyme with vaccine-induced antibodies could potentially cause worm death. The serpin (Bm1_09775) also deserves special mention because of prior work that has been done using protease inhibitors as vaccine candidates in filariasis. The cystatin and serpin protease inhibitors are thought to aid the worm by preventing host proteolytic enzymes from digesting the parasite, and it has been previously hypothesized that these types of proteins may make good vaccine candidates [47, 48]. In a mouse model of onchocerciasis, vaccination with cystatin adsorbed to alum provided 34% protection [49]. However, vaccination with helminth derived proteolytic inhibitors is not always protective [48, 50].

Although this study focused on finding "hidden" vaccine candidates within the digestive tract, it is important to note that these proteins may also be drug targets. Further, we hope to find a single vaccine or drug that can protect against all of the human filarial pathogens. An intriguing aspect of this research is the possibility of producing a single vaccine that can protect against all of the human filarial pathogens. As such, we evaluated whether any of the proteins in S1 Table had high homology across all of the major human filarial pathogens (*W*. *bancrofti*, *O*. *volvulus*, and *Loa loa*) as well as *Dirofilaria immitis*. *D*. *immitis*, the dog heartworm, was included in this analysis to increase the potential benefits of vaccine development. There are significant hurdles to overcome in developing a human vaccine, and it could be advantageous to utilize a vaccine that may potentially also protect against dog heartworm. While a vaccine against this pathogen would certainly be beneficial in its own right, it would also provide significant proof of concept for moving toward human trials. Of the 27 proteins in Table 4, 15 have a sequence of significant homology against all of the filarial pathogens and could be studied as possible pan-filarial vaccines. Importantly 2 proteases (MGC84665 protein related, Bm1_11005 and reprolysin, Bm1_53050) are among these 15 proteins.

While not hidden antigens, helminth excretory-secretory products have been highly studied both as vaccine candidates and for their ability to modulate host immune responses [51–54]. Characterizations of ES products of *B*. *malayi* have previously been performed [5], yet it has never been clear which organ within the worm produces each ES product. ES products can be derived from the worm's surface coat or be secreted or excreted from various orifices including the secretory pore, mouth, anus, and vulva [55, 56]. The structures within the worm that are associated with the production of these ES products are the pharyngeal gland, secretory glands within the lateral cords, and possibly the digestive and reproductive tracts. Although this study does not determine the exact location of production of where individual ES products are made, it does help to narrow down the location of production of some specific ES products to an anatomic fraction. Of the 227 adult female ES products, we identified 159 (70%) within this study. The majority of these proteins were found throughout all three anatomic fractions, which is not surprising considering that these proteins by definition would not be contained within their anatomic origin. For this reason, we focused our analysis on the proteins that were present to higher extent within one anatomic fraction compared to the others. We found that 30 (13%) of these proteins were enriched within the reproductive tract compared to the other fractions. Identification of Juv-p120 (Bm1_18010) as an enriched protein within the reproductive tract is notable as this protein is a well known adult female ES product which may be involved in regulating host immune responses and improving microfilarial survival [57]. However the role of many of the other reproductive tract enriched ES products are less clear. Many of the proteins, such as the von willebrand A domain containing protein (Bm1_27495), are known to have specific binding or signaling functions, but their true role in parasite physiology is unclear. Additionally, many of the proteins are hypothetical proteins that need further study. Similarly, 3 of the 4 digestive tract enriched ES products were hypothetical, while the last was a proteolytic enzyme (Papain family cysteine protease containing protein, Bm1_18805). In contrast to this the ES products enriched within the body wall were all named, and conform to our understanding of the body wall. These include a cuticular protein (Bm1_40465), 2 enzymes that protect from oxidation (cuticular glutathione peroxidase Bm1_40465, and peptide methionine sulfoxide reductase Bm1_10795) as well as many muscular proteins. Protein unc-22 (Bm1_39425) is a protein associated with the A band and appears to have roles in both muscular arrangement and regulation of contraction, as *C*. *elegans* with mutations in unc-22 display disordered muscular growth and muscular twitching [58]. Additionally, the prion-like protein (Bm1_57640) and immunoglobulin-i protein (Bm1_12515) are both similar to the protein Kettin, which is associated with muscular arrangement and may provide stability to the myofibrils during contraction [59].

In conclusion, we have detailed the proteins found within major anatomic fractions of *B*. *malayi*, including the digestive tract, body wall, and reproductive tract. The results suggest that the digestive tract of adult filarial worms likely plays a role in digestion and absorption, and may have other physiologic functions that have not yet been characterized. Further, we have identified 15 vaccine candidates from the *B*. *malayi* digestive tract that could be protective against all major filarial pathogens of humans, and several other intestinal proteins that could have protective efficacy as vaccines against the causative agents of lymphatic filariasis or river blindness.

## Supporting Information

S1 Dataset Proteins identified in the digestive tract, reproductive tract, and body wall of *B*. *malayi*. (XLSX)

S2 Dataset *Wolbachia* proteins identified in the digestive tract, reproductive tract, and body wall of *B*. *malayi*. (XLSX)

S1 Fig Histologic sections of adult female *Brugia malayi* (400x) stained with hematoxylin and eosin. M = Mouth, Pc = pseudocoelom, UT = uterine tubes, Int = itestines, Ov = Ovaries.
(TIF)

S2 Fig Association of immunological (A) and cytoskeletal (B) proteins with the body wall of the adult female *Brugia malayi* as measured by GSEA. P-value<0.001 and = 0.002 respectively. The enrichment score is represented by the green lines. Proteins were rank ordered according to their NSAF values within the body wall, and are depicted in the heat map (red = more abundant, blue = less abundant). Black vertical lines represent each of the proteins associated with proteins of immunological interest (top) and cytoskeletal proteins (bottom) function. D = Digestive tract, R = Reproductive tract, and B = Body wall.
(TIF)

S3 Fig Association of transcription (P<0.001) (A) and nuclear regulation proteins (P = 0.002) (B) with the reproductive tract of adult female *B*. *malayi*. The enrichment score is represented by the green line. Proteins were rank ordered according to their number of spectral counts within the reproductive tract, and are depicted in the heat map (red = more abundant, blue = less abundant). Black vertical lines represent each of the proteins associated with transcription (top) and nuclear regulation (bottom). D = Digestive tract, R = Reproductive tract, and B = Body wall.
(TIF)

S4 Fig Cross section of adult female *B*. *malayi* stained with DAPI (Blue) and Actin (Green). (TIF)

S1 Table Blast-P of digestive tract enriched non-mitochondrial proteins with transmembrane domains against *W*. *bancrofti*, *O*. *volvulus*, *L*. *loa*, *D*. *immitis*, and *H*. *sapiens*. The parameter Query cov. refers to the span of amino acids in the query sequence that aligns with the target sequence producing significant alignment. %Ident is the percentage of amino acids within the query coverage identical to query sequence.
(XLSX)

S2 Table Digestive tract enriched ES products. * Protein was only identified within the digestive tract.
(DOCX)

S3 Table Body wall enriched ES products. * Protein was only identified within the body wall.
(DOCX)

S4 Table Reproductive tract enriched ES products. * Protein was only identified within the reproductive tract.
(DOCX)

S5 Table Digestive tract enriched proteins rank ordered by abundance (NSAF). *specific to the digestive tract
(XLSX)

S6 Table Body wall enriched proteins rank ordered by abundance (NSAF). *specific to the body wall
(XLSX)

S7 Table Reproductive tract enriched proteins rank ordered by abundance (NSAF). *Specific to the reproductive tract.
(XLSX)
