# Supplementary figures and images for: A Proteomic Analysis of the Body Wall, Digestive Tract, and Reproductive Tract of Brugia malayi
Source: PLoS Negl Trop Dis. 2015 Sep 14;9(9):e0004054. doi: 10.1371/journal.pntd.0004054 (PMC4569401; doi:10.1371/journal.pntd.0004054)

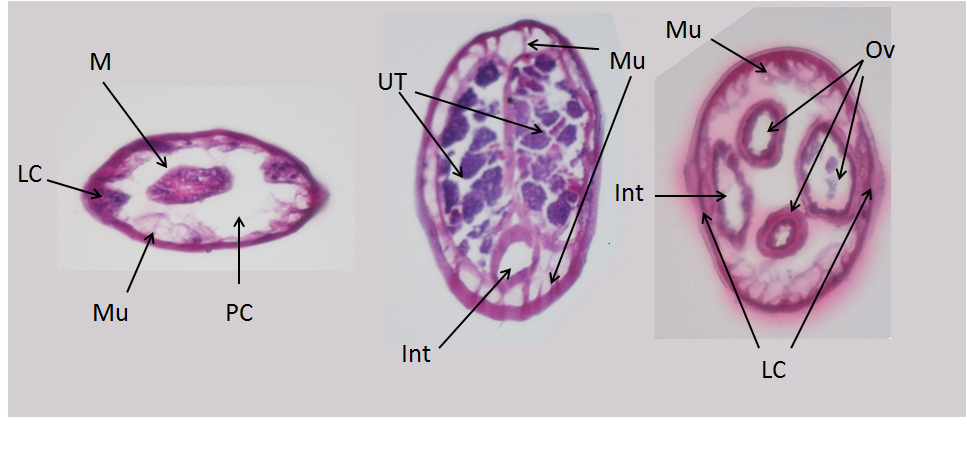

Supplement: S1 Fig — M = Mouth, Pc = pseudocoelom, UT = uterine tubes, Int = itestines, Ov = Ovaries. (TIF) [file pntd.0004054.s003.tif]

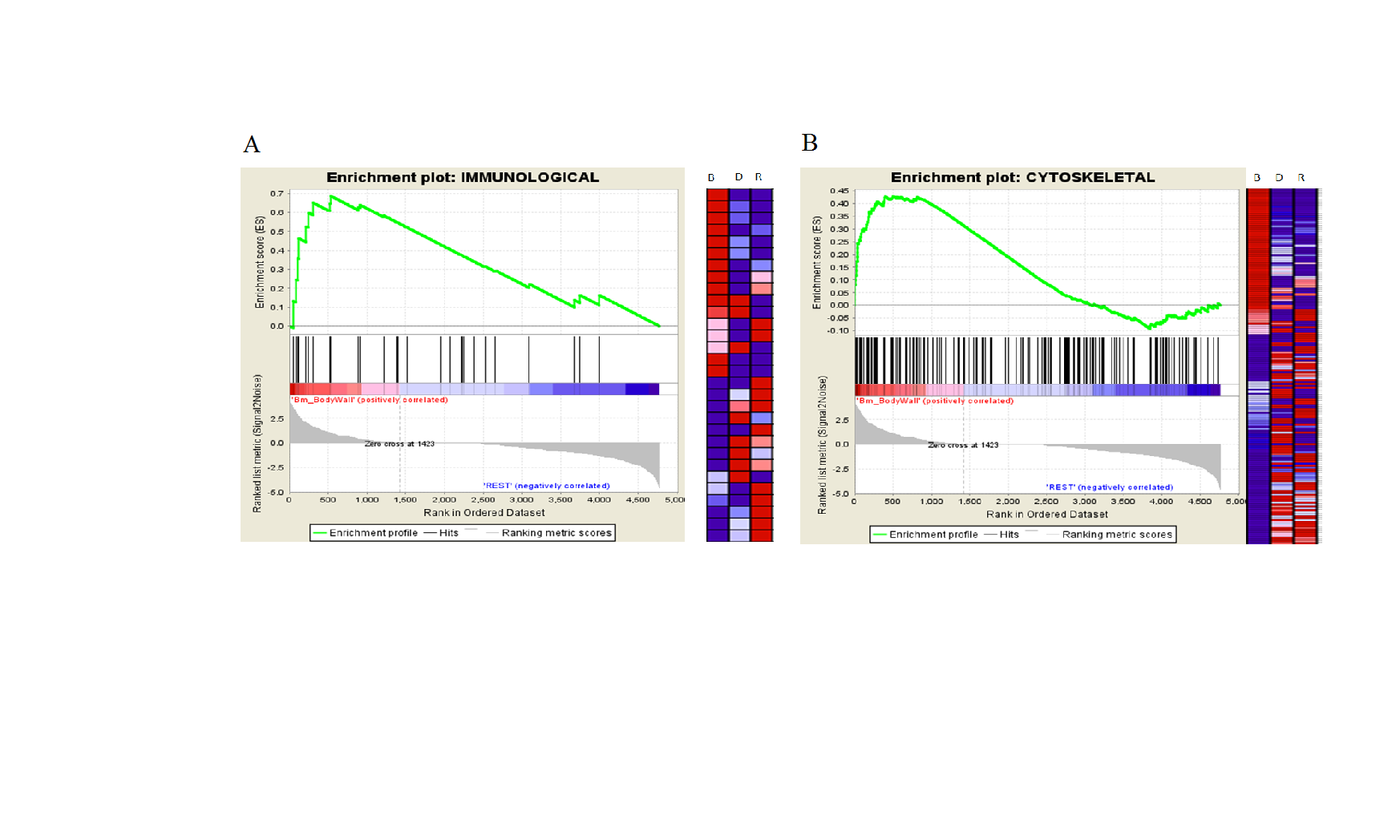

Supplement: S2 Fig — P-value<0.001 and = 0.002 respectively. The enrichment score is represented by the green lines. Proteins were rank ordered according to their NSAF values within the body wall, and are depicted in the heat map (red = more abundant, blue = less abundant). Black vertical lines represent each of the proteins associated with proteins of immunological interest (top) and cytoskeletal proteins (bottom) function. D = Digestive tract, R = Reproductive tract, and B = Body wall. (TIF) [file pntd.0004054.s004.tif]

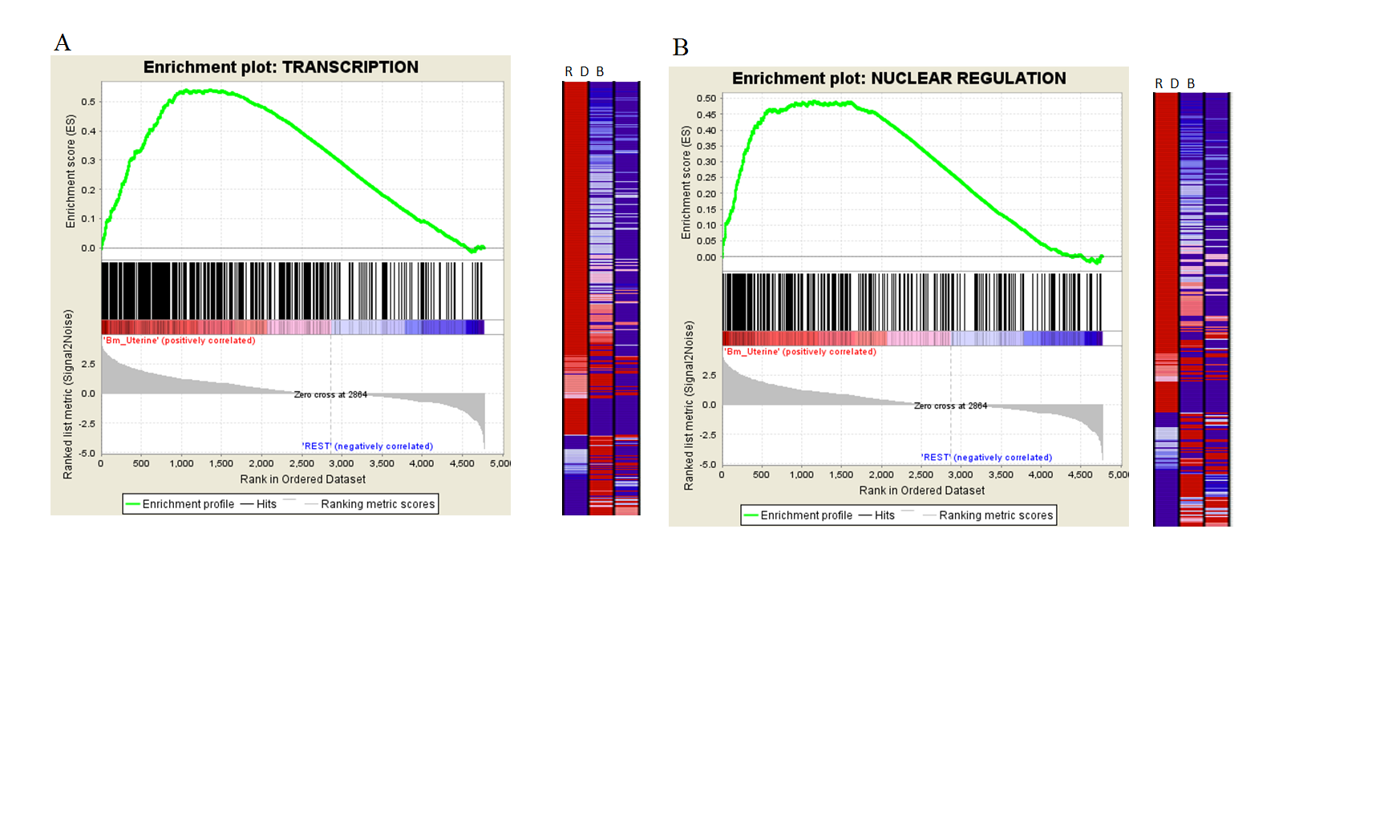

Supplement: S3 Fig — The enrichment score is represented by the green line. Proteins were rank ordered according to their number of spectral counts within the reproductive tract, and are depicted in the heat map (red = more abundant, blue = less abundant). Black vertical lines represent each of the proteins associated with transcription (top) and nuclear regulation (bottom). D = Digestive tract, R = Reproductive tract, and B = Body wall. (TIF) [file pntd.0004054.s005.tif]

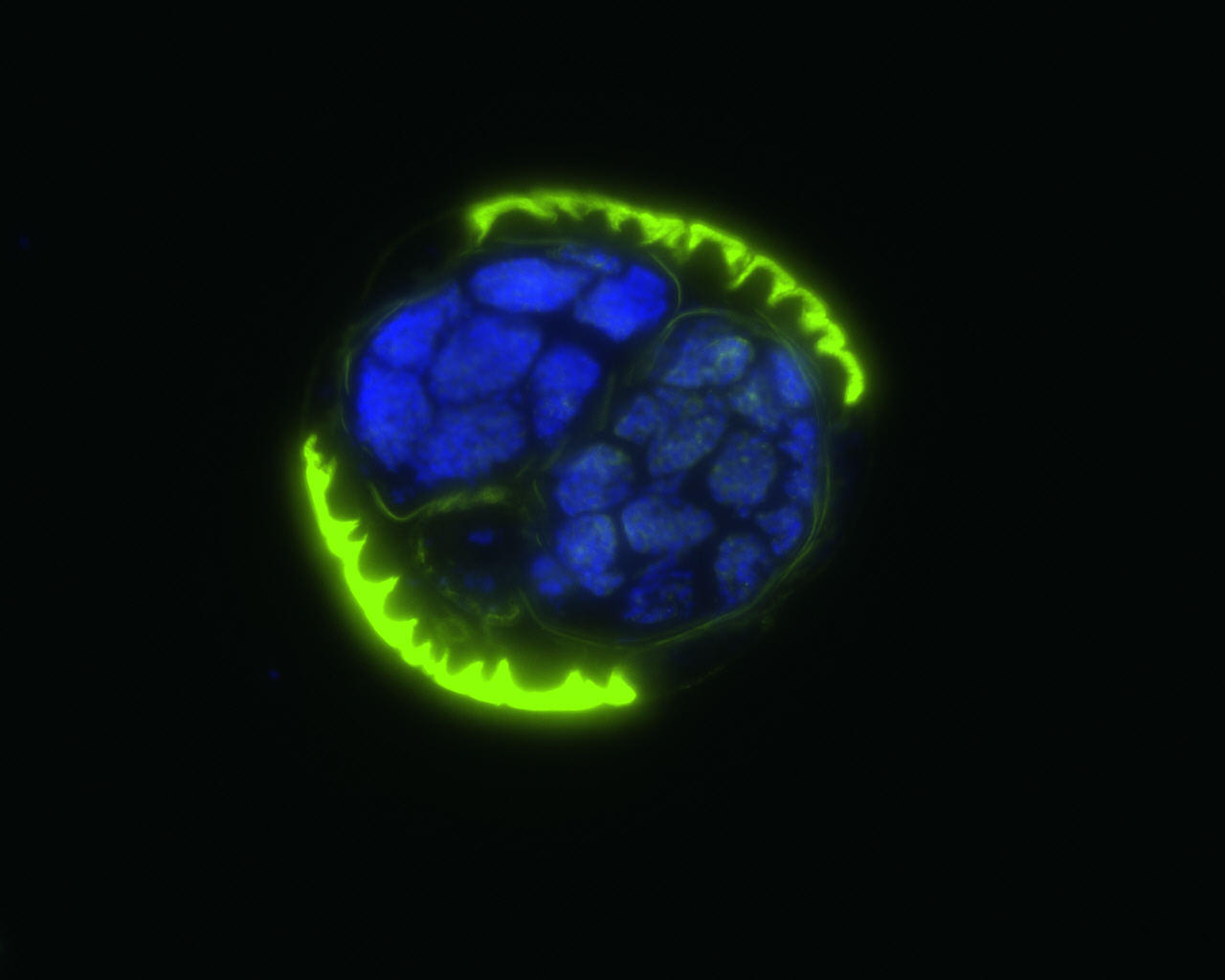

Supplement: S4 Fig — (TIF) [file pntd.0004054.s006.tif]
