# Supplementary material for: A Proteomic Analysis of the Body Wall, Digestive Tract, and Reproductive Tract of Brugia malayi
Source: PLoS Negl Trop Dis. 2015 Sep 14;9(9):e0004054. doi: 10.1371/journal.pntd.0004054 (PMC4569401; doi:10.1371/journal.pntd.0004054)
Supplement: S2 Table — * Protein was only identified within the digestive tract. (DOCX) [file pntd.0004054.s008.docx]

|  |  | Digestive Tract | |
| --- | --- | --- | --- |
| ID | Name | NSAF | NSAF enrichment |
| Bm1_18805 | Papain family cysteine protease containing protein | 4.4E-04 | * |
| Bm1_07875 | CONSERVED HYPOTHETICAL PROTEIN | 1.1E-04 | 4.42 |
| Bm1_20460 | hypothetical protein | 8.0E-05 | 2.95 |
| Bm1_33655 | HYPOTHETICAL 65.5 KDA TRP-ASP REPEATS CONTAINING PROTEIN F02E8.5 INCHROMOSOME X.-RELATED | 5.0E-05 | 2.05 |
