# Supplementary material for: A Proteomic Analysis of the Body Wall, Digestive Tract, and Reproductive Tract of Brugia malayi
Source: PLoS Negl Trop Dis. 2015 Sep 14;9(9):e0004054. doi: 10.1371/journal.pntd.0004054 (PMC4569401; doi:10.1371/journal.pntd.0004054)
Supplement: S3 Table — * Protein was only identified within the body wall. (DOCX) [file pntd.0004054.s009.docx]

|  |  | Body wall | |
| --- | --- | --- | --- |
| Pub_Locus | Name | Abundance (NSAF) | NSAF Enrichment |
| Bm1_13015 | Nematode cuticle collagen N-terminal domain containing protein | 6.1E-03 | 3.05 |
| Bm1_50805 | Myosin tail family protein | 4.7E-03 | 4.00 |
| Bm1_40465 | Cuticular glutathione peroxidase precursor, putative | 4.5E-03 | 2.59 |
| Bm1_39425 | protein unc-22, putative | 8.8E-04 | 7.42 |
| Bm1_26690 | Prion-like-, putative | 8.0E-04 | 4.73 |
| Bm1_12515 | Immunoglobulin I-set domain containing protein | 4.5E-04 | 7.68 |
| Bm1_45145 | Ryanodine Receptor TM 4-6 family protein | 1.4E-04 | 3.67 |
| Bm1_10795 | Peptide methionine sulfoxide reductase family protein | 9.6E-05 | * |
