# Supplementary material for: A Proteomic Analysis of the Body Wall, Digestive Tract, and Reproductive Tract of Brugia malayi
Source: PLoS Negl Trop Dis. 2015 Sep 14;9(9):e0004054. doi: 10.1371/journal.pntd.0004054 (PMC4569401; doi:10.1371/journal.pntd.0004054)
Supplement: S4 Table — * Protein was only identified within the reproductive tract. (DOCX) [file pntd.0004054.s010.docx]

|  |  | Reproductive Tract | |
| --- | --- | --- | --- |
| Pub_Locus | Name | Abundance (NSAF) | NSAF Enrichment |
| Bm1_29260 | 60S ribosomal protein L34, putative | 4.6E-04 | 2.4 |
| Bm1_20440 | Biotin/lipoate A/B protein ligase family protein | 4.5E-04 | 2.8 |
| Bm1_43080 | hypothetical protein | 3.7E-04 | 2.1 |
| Bm1_02485 | Potential global transcription activator SNF2L, putative | 2.5E-04 | 2.6 |
| Bm1_16970 | hypothetical protein | 1.8E-04 | 2.7 |
| Bm1_16690 | Aspartyl aminopeptidase, putative | 1.6E-04 | 3.4 |
| Bm1_18010 | excretory/secretory protein Juv-p120 precursor-related | 1.4E-04 | 3.4 |
| Bm1_21390 | RNA binding protein, putative | 1.3E-04 | 2.0 |
| Bm1_48000 | RAS FAMILY PROTEIN | 1.1E-04 | 2.7 |
| Bm1_12225 | G-patch domain containing protein | 1.0E-04 | 3.9 |
| Bm1_03520 | Kunitz/Bovine pancreatic trypsin inhibitor domain containing protein | 9.9E-05 | 2.8 |
| Bm1_02770 | Galactosyltransferase family protein | 8.5E-05 | 2.6 |
| Bm1_48025 | Alpha-catulin, putative | 7.5E-05 | 3.5 |
| Bm1_41495 | Gex interacting protein protein 4, isoform c-related | 7.2E-05 | 7.0 |
| Bm1_18480 | HYPOTHETICAL PROTEIN | 7.2E-05 | * |
| Bm1_46460 | 26S PROTEASOME REGULATORY CHAIN 4, PUTATIVE | 6.4E-05 | 5.4 |
| Bm1_49790 | tRNA modification GTPase TrmE family protein | 5.1E-05 | 2.0 |
| Bm1_27495 | von Willebrand factor type A domain containing protein | 4.9E-05 | 2.1 |
| Bm1_46930 | HYPOTHETICAL PROTEIN | 4.4E-05 | 2.1 |
| Bm1_21025 | hypothetical protein, conserved | 4.3E-05 | 3.4 |
| Bm1_25670 | hypothetical protein | 4.0E-05 | * |
| Bm1_41650 | hypothetical protein | 3.2E-05 | 4.1 |
| Bm1_54890 | RNA recognition motif. | 3.0E-05 | 2.0 |
| Bm1_16685 | hypothetical protein | 2.7E-05 | 2.7 |
| Bm1_40395 | Phosphatidylinositol 3- and 4-kinase family protein | 2.6E-05 | 4.3 |
| Bm1_00750 | RhoGEF domain containing protein | 1.9E-05 | 3.4 |
| Bm1_25450 | Formin Homology 2 Domain containing protein | 1.8E-05 | * |
| Bm1_05305 | HYPOTHETICAL PROTEIN, CONSERVED | 1.7E-05 | * |
| Bm1_42420 | Eye-specific diacylglycerol kinase, putative | 1.6E-05 | * |
| Bm1_17115 | conserved hypothetical protein | 9.8E-06 | * |
